# EXAMINATION OF THE DIGIAGE CYBER-SENIORS PILOT STUDY FOR OLDER ADULTS FROM LOWER INCOME COMMUNITIES

**DOI:** 10.1093/geroni/igac059.2197

**Published:** 2022-12-20

**Authors:** Skye Leedahl, Alexandria Capolino, Melanie Brasher, Kristin Fratoni-Souza, Erica Estus

**Affiliations:** University of Rhode Island, Kingston, Rhode Island, United States; University of Rhode Island, Kingston, Rhode Island, United States; University of Rhode Island, Kingston, Rhode Island, United States; University of Rhode Island, Kingston, Rhode Island, United States; University of Rhode Island, Kingston, Rhode Island, United States

## Abstract

The University of Rhode Island (URI) team implemented a pilot program designed to bridge the digital divide between older adults and younger generations. Through this project, the goal was for older Rhode Islanders to become digitally literate by engaging them in a formal program that provides digital devices (i.e., Apple iPads), connectivity (i.e., internet connection through HotSpots), and training by supervised and trained university student mentors. This project specifically worked to promote social and economic equity by recruiting participants from lower-income communities and areas hit hardest by the COVID-19 pandemic, including those who speak English and Spanish. The final sample (N=124) for the study was primarily female (79%) and low income (82%). They were 41% Non-White and 23% Spanish speaking with 23% having a college degree or more. We conducted preliminary analysis on the first 38 participants who completed both a pre- and post-survey. Based on these analyses, the program participants have shown statistically significant improvements in frequency of tablet use (p<.05), digital competency across all questions (p<.05), quality of life (p<.05), and social isolation (p<.05). In addition, the loneliness and depression questions/variables are trending in the expected direction, so it is possible that with a more robust sample size we may see significant improvement in these areas as well. This presentation will describe the key elements of the pilot intervention and explain the findings from the full sample. The presentation will also discuss the plan for state-wide implementation of the program.

